# Decoding the mechanism behind MCL-1 inhibitors: A pathway to understanding MCL-1 protein stability

**DOI:** 10.18632/oncotarget.28440

**Published:** 2023-06-21

**Authors:** Shady I. Tantawy, Natalia Timofeeva, Ana Hernandez, Aloke Sarkar, Varsha Gandhi

**Keywords:** AMG-176, AZD5991, B-cell leukemia, MCL-1 inhibitor, MCL-1 protein

***Commentary on:** Mechanisms of MCL-1 protein stability induced by MCL-1 antagonists in B-cell malignancies. Clinical Cancer Res. 2023; 29:446–57. https://doi.org/10.1158/1078-0432.ccr-22-2088. [PubMed]*

The diversity of BH3 proteins can be broadly characterized as pro-survival (BCL-2, MCL-1, BCL-xL, Bfl-1/A1, Bcl-w, Bcl-b) or pro-apoptotic (Bax, Bak; and Bim, Puma, BID, BAD, BIK, HRK, BMF, Noxa). Pro-survival proteins function by sequestering the pro-apoptotic proteins, thus preventing the initiation of an apoptotic cascade. However, overexpression of pro-survival proteins can help in tumorigenesis and drug resistance, making them an attractive target for inducing apoptosis in cancer cells [1]. The successful development of BCL-2 inhibitor (venetoclax), currently approved for chronic lymphocytic leukemia (CLL) and acute myeloid leukemia (AML), encouraged the development of inhibitors that target other antiapoptotic proteins, particularly Myeloid Leukemia 1 (MCL-1).

MCL-1 is among the top genes amplified in several cancers and is implicated in cancer progression, drug resistance and poor prognosis. It protects cancer cells from apoptosis and decreases their sensitivity to targeted agents or chemotherapeutics [2–6]. MCL-1 preferentially binds to Bak and Noxa, thus inhibiting the release of cytochrome C and activation of the apoptotic cascade. Besides, MCL-1 performs other functions including DNA repair, cell cycle regulation, mitophagy, autophagy, metabolism, and cellular senescence. MCL-1 is critical for embryonic development and associated with the survival of many cells, including the nervous system, T/B lymphocytes and cardiomyocytes [7].

MCL-1 is a short-lived protein (less than one hour), and its expression is regulated at transcriptional, post-transcriptional, translational, post-translational and proteasomal degradation levels. The stability and degradation of MCL-1 is controlled by serine (Ser)/threonine (Thr) phosphorylation at specific residues. ERK-mediated phosphorylation at Thr^163^ residue enhances MCL-1stability, while GSK3β mediated Ser^159^ phosphorylation leads to MCL-1 degradation. Interaction of MCL-1 with other proapoptotic proteins such as Bim and Noxa also determines its fate; Noxa was shown to degrade MCL-1 while Bim stabilizes MCL-1 protein. Multiple E3 ligases (Mule, β-TRCP) ubiquitinate and degrade MCL-1, a process that can be reversed by deubiquitinases (DUBs) (USP9x, KU-70) [8].

Preclinical investigations of several clinically relevant MCL-1 inhibitors (MCL-1i) in hematologic malignancies demonstrated an increase in MCL-1 protein accumulation following treatment with these MCL-1i, including Novartis’ S63845 (NCT02979366) [9], AstraZeneca’s AZD5991 (NCT03218683) [10, 11] and Amgen’s AMG-176 (NCT02675452) [12, 13]. However, the mechanism of this protein accumulation is largely unknown. To address this knowledge gap, Tantawy et al. explored underlying molecular mechanisms that contribute to MCL1i-induced MCL-1 protein accumulation and its implications [14].

This study revealed a complex and multifaceted nature of MCL-1 protein accumulation in B-cell malignancies upon treatment with MCL-1i (AMG-176 and AZD5991). They showed that the observed MCL-1 protein accumulation was unrelated to transcriptional activation of *Mcl-1* gene but due to increased stability. However, this effect was reversible following withdrawal of MCL-1i, indicating a direct effect of the drug on MCL-1 protein stability. Using protein-protein interaction studies, immunoprecipitation (IP) and co-IP experiments, Tantawy et al. [14] showed that MCL-1i-induced defective ubiquitination of MCL-1 protein. This indicates that the upregulation of MCL-1 protein is not due to direct proteasomal inhibition, but rather due to interference with the ubiquitination and/or enhancing de-ubiquitination of MCL-1.

**Figure 1:**
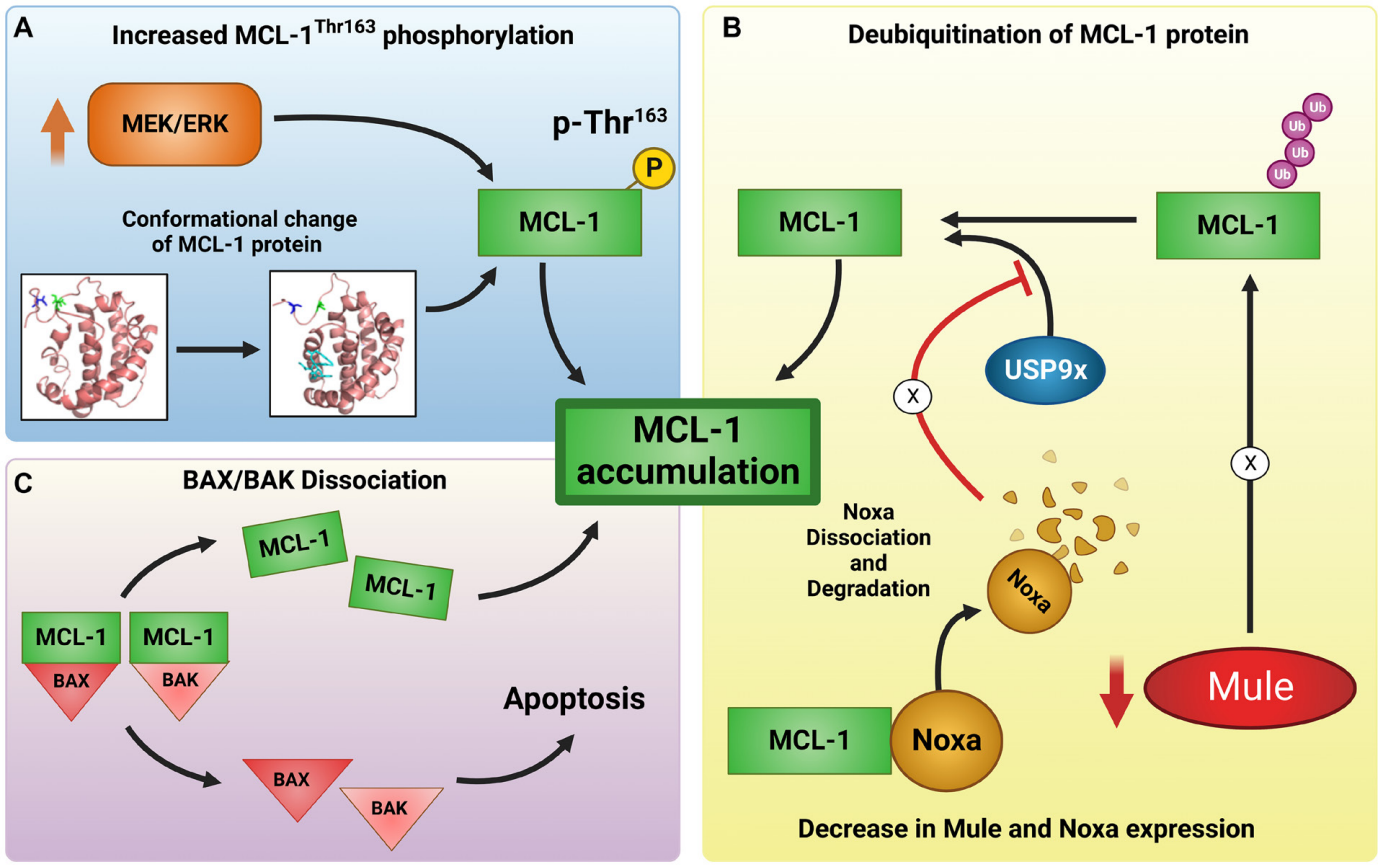
Mechanisms of MCL-1 inhibitor-induced MCL-1 protein upregulation, stability, and induction of apoptosis. Three major pathways (**A**–**C**) have been demonstrated for upregulation of MCL-1 protein after treatment with MCL-1 inhibitors. (A) The MCL-1 protein stability is facilitated in part following binding with the MCL-1 inhibitors leading to a conformational change in the protein that enhance MCL-1^Thr163^ phosphorylation by upstream MEK/ERK signaling pathway. (B) MCL-1 inhibitors treatment enhanced DUB activity and induced Noxa dissociation from MCL-1, followed by rapid Noxa degradation leading to MCL-1 stability through potentiating USP9x: MCL-1 interaction. Additionally, MCL-1 inhibitors reduce the levels of the E3 ligase Mule, resulting in defective ubiquitination of MCL-1. The net effect is seen in an increased stability of the MCL-1 protein. (C) MCL-1 inhibitor binding to MCL-1 protein induces MCL-1 dissociation from BAX/BAK pro-apoptotic protein complex, facilitating their oligomerization resulting in induction of apoptosis. Black arrows indicate potentiating effect, red arrows indicate inhibitory effect. X marks indicate disruption of the normal pathway.

Tantawy et al. [14] further addressed the mechanisms underlying the decreased ubiquitination of MCL-1 protein and showed that MCL-1i increased MEK/ERK-mediated Thr^163^ phosphorylation, leading to conformation change of MCL-1 protein. This in turn increases the surface accessible area to Thr^163^ residue, thereby contributing to MCL-1 stability and accumulation. This observation was further supported by abolishing MCL-1 Thr^163^ phosphorylation by using MCL-1-T163A phospho-mutant or direct inhibitor, trametinib, of upstream MEK/ERK pathway. In either approach, authors showed only partial effect of MCL-1i- induced reversal of MCL-1 protein upregulation in a cell type specific manner, suggesting the presence of other underlying mechanisms (Figure 1A).

The group [14] further explored the effect of MCL-1i on the ubiquitination and de-ubiquitination of MCL-1. Using cell free *in vitro* ubiquitination/deubiquitination assays, the authors showed that MCL-1i directly enhanced DUB activity on MCL-1 protein, possibly by orienting MCL-1 to a state/conformation that does not favor ubiquitination and prefers deubiquitination by USP9x. This is further enhanced by the fact that MCL-1i disrupted MCL-1: Noxa interaction followed by rapid Noxa degradation and also associated with a transient decrease in the E3 ligase Mule protein level. Noxa normally degrades MCL-1 by favoring Mule-MCL-1 interaction and by opposing USP9x-MCL-1 interaction, thereby, its downregulation will contribute to MCL-1 protein stability. The contribution of DUBs in the observed MCL-1 stability was further re-affirmed by these authors using a global DUB inhibitor (WP1130), that rescued the MCL-1 upregulation induced by the MCL-1i (Figure 1B). Interestingly, the two MCL-1i tested, AMG-176 and AZD5991, showed distinct effects on MCL-1 ubiquitination in a cell free *in vitro* ubiquitination assay, with AMG-176 enhancing while AZD5991 inhibiting it. Although the authors did not explore this unique observation further in detail, they predicted that it could be due to the binding difference between AMG-176 and AZD5991 to the BH3 binding grove of MCL-1 (AMG-176 lacks the salt bridge formation with Arg^263^ of MCL-1) or due to different effect on the QRN motif of MCL-1 that may impact MCL-1 stability and degradation. Authors identified MCL-1i-induced important molecular changes particularly decrease in both Mule and Noxa protein levels, which may have implications in MCL-1i-induced cardiotoxicity. This is consistent with reports linking the critical role of Mule and Noxa in cardiotoxicity in murine models [15, 16].

Despite high accumulation of MCL-1 protein after interaction with MCL-1i, there was induction of apoptosis. Authors explain this conundrum; MCL-1i disrupted both BAK and BAX interaction with MCL-1 which may be responsible for apoptosis (Figure 1C). Interestingly, AZD5991 is more potent than AMG-176 in inducing apoptosis in either cell lines or in primary CLL lymphocytes, the reason for this difference is unknown.

Taken together, the study [14] provides novel insights into the regulation of MCL-1 expression by MCL-1i, associated molecular changes and their implications. They demonstrated that MCL-1i upregulated MCL-1 protein through direct and indirect mechanisms. MCL-1i led to enhanced DUB activity on MCL-1 protein, disruption and downregulation of Noxa as well as a transient decrease in Mule expression. The net effect was seen in MCL-1 protein stability. These molecular changes require further experiments to explore the potential role in MCL-1i- induced cardiotoxicity. Authors did not evaluate S63845 but identified differences between AMG-176 and AZD5991 in terms of potency. Collectively, these findings may have important implications for the development of next-generation MCL-1i with improved efficacy and safety profiles.
